# Effect of diode laser irradiation on the bond strength of polymerized non-simplified adhesive systems after 12 months of water storage

**DOI:** 10.1590/1678-7757-2018-0126

**Published:** 2018-12-10

**Authors:** Giovanna Speranza Zabeu, Rafael Massunari Maenossono, Caren Roberta Scarcella, Letícia Ferreira Freitas Brianezzi, Regina Guenka Palma-Dibb, Sérgio Kiyoshi Ishikiriama

**Affiliations:** 1 Universidade de São Paulo, Faculdade de Odontologia de Bauru, Departamento de Dentística, Endodontia e Materiais Odontológicos, Bauru, São Paulo, Brasil; 2 Fundação Municipal de Educação e Cultura de Santa Fé do Sul, Departamento de Dentística, Santa Fé do Sul, São Paulo, Brasil; 3 Instituto Chaddad de Ensino, Faculdade do Sudoeste Paulista, Departamento de Dentística, Avaré, São Paulo, Brasil; 4 Universidade de São Paulo, Faculdade de Odontologia de Ribeirão Preto, Departamento de Odontologia Restauradora, Ribeirão Preto, São Paulo, Brasil

**Keywords:** Dentin bonding agents, Lasers, Tensile strength

## Abstract

**Objectives:**

The aim of this *in vitro* study was to evaluate the bonding strength of non-simplified dentin bonding systems (DBS) to dentin irradiated with a diode laser (970 nm) immediately and after 12 months of water storage following either primer or bond application.

**Material and methods:**

The experimental design included three different factors: DBS type [Adper^TM^ Scotchbond Multipurpose (MP) and Clearfil™ SE Bond (CSE)], irradiation [without irradiation - control (C), irradiation after primer application (AP), and irradiation after bond application (AB)], and time [initial (I) and after 12 months of water storage (12 m)]. Sixty sound human third molars (n = 10) were obtained, and their flat occlusal dentin areas were prepared and standardized. Laser irradiation was performed in the contact mode perpendicular to the dental surface over an automatically selected scanning area at a pulse energy of 0.8 W, frequency of 10 Hz, and energy density of 66.67 J/cm^2^. After 7 days of treatment, the specimens were cut, and half of them were subjected to microtensile testing (500 N/0.05 mm/min), whereas the remaining sticks were examined after 12 months of water storage. The obtained data were analyzed by three-way analysis of variance (ANOVA) followed by a Tukey test (p<0.05). The observed fracture modes were investigated using a portable digital microscope with a magnification of 40x.

**Results:**

Among the utilized DBS, MP generally exhibited higher bond strengths, but did not always differ from CSE under similar conditions. The irradiation factor was statistically significant only for the MP/AB groups. After 12 months of storage, all groups demonstrated a significant reduction in the bond strength, whereas the results of fracture analysis showed a predominance of the adhesive type.

**Conclusions:**

The laser treatment of non-simplified DBS was not able to stabilize their bonding characteristics after 12 months.

## Introduction

Polymeric restorative procedures performed on dental substrates rely essentially on the ability of the currently available dentin bonding systems (DBS) to promote interaction with demineralized dentin via mechanical imbrication or chemical bonding. ^1^ ^-^ ^3^ Margin restorations on enamel typically exhibit good adhesion characterized by high longevity, which is comparable with that of amalgam restorations. ^4^ However, the dentin substrate forms a relatively weak bond with the resin due to its complexity and fragility, ^5^ causing clinical failures related to marginal infiltration and aesthetics. ^6^

Different strategies have been proposed to improve the long-term dentin bonding strength. ^7^ ^-^ ^11^ Some of them focused on the obtention of more hydrophobic polymer resins and/or their ability to chemically interact with dentin, while other works aimed at reinforcing collagen fibers. One of such approaches combined the laser technology with DBS which has been considered promising, since high values of their bonding strengths were observed in laboratory studies. ^12^ ^-^ ^14^

Maenosono, et al. ^14^ (2015) found that noticeably higher bond strengths were achieved when simplified DBS were irradiated with a diode laser (970 nm). This phenomenon was attributed to the solvent evaporation process enhanced by laser irradiation due to either the existence of laser-solvent interactions or high temperature. However, no studies on non-simplified DBS were performed to validate this assumption. Since the use of such systems involves multiple steps including primer application, the same rationale can be applied in their case because the primers of nonsimplified DBS also contain solvents. In the majority of previous studies, DBS were irradiated by various types of lasers after adhesive application as had been initially suggested by Gonçalves, de Araujo, and Damião ^13^ (1999).

In the present work, it is hypothesized that the irradiation of DBS after primer application can increase their bonding strength due to the presence of solvent in the primer. Therefore, to achieve a better understanding of the mechanism of laser-solvent interactions, a diode laser was used at different stages of the bonding process. The objective of this study was to estimate the bonding strengths of non-simplified DBS to dentin irradiated with a diode laser after the primer or bond application.

## Material and methods

### Experimental design

The conducted *in vitro* study involved the following factors: 1) two types of DBS [Adper™ Scotchbond Multi-Purpose (MP) and Clearfil™ SE Bond (CSE)], 2) three types of irradiation [no irradiation - control (C), laser irradiation after primer application (AP), and laser irradiation after bond application (AB)], and 3) two different times [initial (I) and after 12 months of water storage (12 m)]. The measured response parameter was the microtensile bond strength. The observed failures were analyzed using a digital microscope with a magnification of 40x.

### Specimen preparation

After the approval of the Ethics Committee of Research on Human Beings (CAAE 310887/4.1.0000.5417), sixty sound human third molars were obtained and randomized into six groups according to the sizes of the exposed dentin areas (n = 10). To expose dentin, the occlusal face was sectioned perpendicular to the long axis of the tooth using a sectioning machine (Isomet™ Low Speed Saw®, Buehler; Lake Bluff, IL, USA) with a water-cooled diamond disc (Extec Corporation; Enfield, CA, USA). Enamel remaining was removed using #320 grit paper (Carbimet Paper Discs, Buehler; Lake Bluff, IL, USA), and a smear layer was simulated with #600 grit paper for 30 s using a polishing machine (Arotec; Cotia, SP, Brazil).

A condensation-cured silicon mold (Zetalabor, Zhermack; São Paulo, SP, Brazil) was positioned centrally on the dentin surface to obtain a standardized area of 36 mm^2^ to ensure effective laser treatment. A white nail polish (Revlon, Revlon International Corp; New York, NY, USA) was applied around the mold to limit the testing area.

### Laser treatment

**Figure 1:**
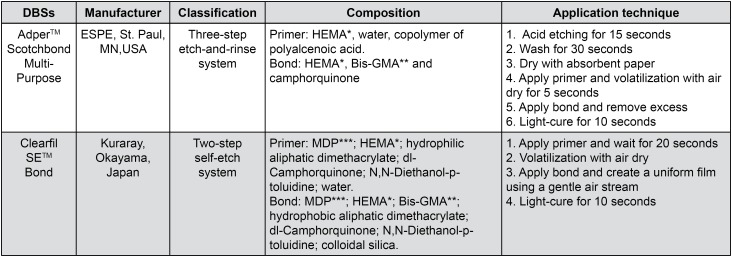
Commercially available systems, classification, composition and application technique * HEMA: 2-hydroxyethyl methacrylate ** Bis-GMA: Bisphenol A diglycidyl methacrylate *** MDP: 10-methacryloyloxydecyl dihydrogen phosphate

Each group received proper treatment according to the manufacturers’ instructions ( Figure 1 ). After the primer or bonding application, a diode laser (SiroLaser, Sirona; Bensheim, HE, Germany) was activated on the substrate surface in the contact mode perpendicular to the dental surface followed by the automatic scanning of the selected area standardized for 30 s using an XY table (BioPDI; São Carlos, SP, Brazil). Laser irradiation was performed at a pulse energy of 80 mJ, frequency of 10 Hz, power of 0.8 W, energy density of 66.67 J/ cm^2^, and total energy of 24 J. All DBS were applied according to the manufacturers’ instructions ( Figure 1 ) and light-cured for 10 s using a light-emitting diode (Blue Star 2, Microdont; São Paulo, SP, Brazil) with an energy density of 1,000 mW/cm^2^. Restorations were performed using Filtek Z250 XT composite (3M ESPE; St Paul, MN, USA) in three consecutive 1.5-mm increments followed by light curing for 10 s after each step.

The prepared specimens were stored in deionized water at a temperature of 37°C for 7 d. After that, they were sectioned perpendicular to the occlusal surface with a low-speed diamond saw (Isomet, Buehler; LakeBluff, IL, USA) to obtain resin-dentin sticks with cross-sectional areas of 0.64 mm^2^. The produced sticks with an average number of 10 *per* group/time were randomly divided into the two times: initial and stored for 12 months. During storage, the sticks were kept in deionized water, which was periodically replaced every 15 d. Sticks with premature failures before the test were counted as 0 MPa.

### Microtensile test

The specimens were tested using a universal testing machine (Instron 3342, Illinois Tool Works; Norwood, IL, USA). The area of each sample was measured with a digital caliper (Digimatic Caliper Absolute, Mitutoyo Corp; Kawasaki, Kanagawa, Japan), and the obtained values were entered into the BlueHill software (BlueHill® Materials Testing Software; Norwood, IL, USA). Afterwards, the specimens were fixed with a cyanoacrylate-based adhesive (Loctite Super Bonder Gel Control, Henkel Ltda; São Paulo, SP, Brazil) in the machine's dispositive (Bencor, Danville Engeneering; Danville, CA, USA). The adhesive interface was positioned perpendicular to the tensile forces generated by the testing machine. Tension was applied at a constant speed of 0.5 mm/min, and the maximum load required to break the specimen was equal to 500 N.

### Failure mode analysis

Each fractured surface was examined with a digital portable microscope (Dino Lite Microscope Plus, AnMo Electronics Corp; New Taipei City, Taiwan, China) to determine a particular type of failure among the following categories: adhesive, cohesive in dentin, cohesive in resin, and mixed.

### Statistical analysis

The obtained data were analyzed statistically using the Statistica software (Statsoft^®^; Tulsa, OK, USA). The assumptions of normal distribution and equality of variances were checked for all variables using the Kolmogorov-Smirnov and Levene tests, respectively. After validating these assumptions, the data were subjected to three-way analysis of variance (ANOVA) and Tukey's test (p<0.05).

### Scanning electronic microscopy (SEM)

Two additional specimens from each group were prepared for scanning electron microscopy (SEM) observations using the same protocol. Briefly, they were placed in stubs, sputter coated with gold, and then examined using an SEM instrument (JSM T220A, JEOL USA; Peabody, MA, USA) at a magnification of ×1,500 ^15^ .

## Results

### Bond strength

The mean values of the measured bond strengths and their standard deviations are summarized in Table 1 . The obtained data revealed statistical significances of the DBS (p=0.017) and time (p<0.0001) factors, but not of the laser factor (p=0.093). Except for the DBS x pretreatment interaction (p = 0.013), there were no significance of interactions among the factors evaluated (p>0.05).

**Table 1:** Mean and standard deviations values (MPa) of bond strength

DBS		Initial	12 months
	Control	46.55±10.69^Aa^	37.65 ±10.66^Ba^
MP	AP	44.34±6.43^Aab^	28.86±16.46^Bab^
	AB	37.20±12.96^Ab^	20.06±16.90^Bb^
	Control	31.47±8.41^Ab^	25.91±10.24^Bb^
CSE	AP	39.62±11.39^Aab^	24.13±10.75^Bab^
	AB	40.10±11.36^Ab^	22.08±11.77^Bb^

Different uppercase letters indicate statistical differences for the same condition (DBS and pretreatment) in different time evaluation

Different lowercase letters indicate statistical differences for the same DBS x pretreatment in the same time

Data from 12 months systematically smaller than the magnitudes measured for the as-prepared specimens under all conditions. In terms of the DBS factor, MP demonstrated the highest bond strength values for all groups and measurement times. Furthermore, MP/AB was the only group of specimens negatively affected by laser irradiation. CSE was not affected by laser irradiation.

The failure mode distributions determined for different sample groups are listed in Table 2 .

**Table 2:** Failure modes distribution (%)

Time	Group	Adhesive	Cohesive in dentin	Cohesive in resin	Mixed
Initial	MP/C	50%	25%	21%	4%
	MP/AP	60%	5%	5%	30%
	MP/AB	67%	28%	5%	0%
	CSE/C	53%	6%	12%	29%
	CSE/AP	55%	4%	18%	23%
	CSE/AB	61%	20%	14%	5%
12 months	MP/C	78%	4%	4%	14%
	MP/AP	73%	9%	9%	9%
	MP/AB	78%	0%	15%	7%
	CSE/C	76%	9%	6%	9%
	CSE/AP	72%	14%	14%	0%
	CSE/AB	75%	5%	9%	11%

### SEM studies

**Figure 2:**
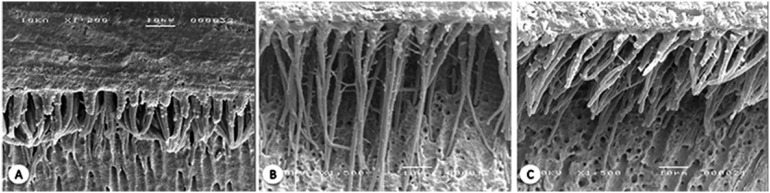
Hybrid layer produced when MP was used. A- control: homogeneous tags are observed. B- AP: Longer tags were observed when the laser was irradiated after primer application. C- AB: Similar observations compared with control group were found when the diode laser was irradiated after bond application

**Figure 3:**
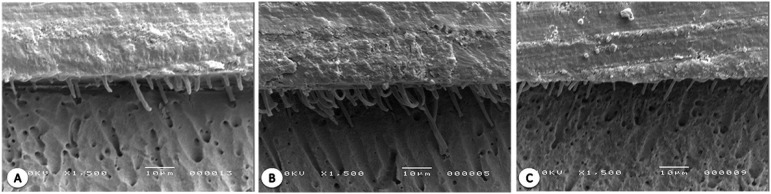
Hybrid layer produced when CSE was used. A- control: thin tags were observed. B- AP: tags were also produced and maintained associated after diode laser application after primer application. C- AB: Similar observation was found compared with control group when the diode laser was applied after bond application

The representative SEM images obtained for each experimental group are shown in Figures 2 and 3 . For MP, notable increases in the amount and thickness of the formed tags were observed after the laser irradiation following primer application. For CSE, the formation of thinner tags occurred for all specimen groups.

## Discussion

Diode lasers exhibit various advantages such as versatility, relatively small dimensions, and low costs that make them particularly useful in clinical applications. Since these lasers are widely utilized in dentistry, ^16^ ^-^ ^19^ expanding the scope of their applicability can provide significant benefits. In several studies, diode lasers were used to improve the performance of DSB. The pioneering work in this area was published by Maenosono, et al. ^14^ (2015) who performed laser irradiation after the application of simplified adhesive systems. In those systems, the primer and solvent were combined with a bonding agent, and the observed effectiveness of laser irradiation was attributed to solvent evaporation. However, in non-simplified systems, the primer and bonding agent are separated, whereas solvent species are present exclusively in the primer. Moreover, mixing monomers and diluents affects not only the bonding dynamics, but also the physical and mechanical properties of the material. ^3^ ^,^ ^20^ ^,^ ^21^

Therefore, in the present work, a high-power laser was used to isolate the solvent and confirm the mechanism proposed in a previous study. ^14^ Furthermore, the bonding of two non-simplified DBS to the dentin substrate was investigated. Because the durability of the bonding interface is an important factor that affects the quality and longevity of polymeric adhesive restorations, different laboratory strategies are utilized to simulate interface aging. In this study, it was achieved via the water storage at a temperature of 37°C for 12 months with frequent water exchange. ^22^

The obtained results showed no differences between the control group and the specimens treated with the diode laser before the application of the primer or bonding agent. The findings of this study do not agree with the data reported in previous works, suggesting that laser irradiation can improve the bonding strength of DBS to dentin. Some authors observed an increase in the bond strength after the laser irradiation of non-polymerized adhesives. ^12^ ^-^ ^14^ Gonçalves, de Araujo and Damião ^13^ (1999) who proposed to use an experimental laser for DBS treatment observed an increase in the bonding strength of the conventional three-step DBS by conducting micro-shear testing. Franke, et al. ^12^ (2006) and Marimoto, et al. ^23^ (2013) also reported increased bond strengths after the irradiation of simplified adhesive systems with an Nd:YAG laser (1064 nm).

Recently, Maenosono, et al. ^14^ (2015) used a laser diode (970 nm) to increase the bonding strengths of simplified adhesive systems. Brianezzi, et al. ^24^ (2017) found that the application of a diode laser could increase the degree of conversion of such systems without negatively affecting their sorption and solubility properties. However, in all these studies, the bonding strengths were measured immediately after sample preparation. Furthermore, the mechanisms of bonding strength improvement are similar for both types of lasers since the effect produced by high-power lasers is based on the increase in temperature.

After performing the initial time analysis, no significant differences were observed for various DBS types or utilized strategies. According to the study conducted by De Munck, et al. ^22^ (2003), non-simplified DBS are less prone to degradation as compared to the simplified systems, and the use of a laser might not be sufficient for improving their performance. These results indicate that the separate use of the primer and bonding agent significantly enhances the DBS performance regardless of the laser application.

The obtained SEM images showed a small increase in the depth of the tags formed only in the MP/AP group. In terms of the DBS type, the bond strengths of the MP groups were higher than those of the CSE groups with respect to the control group. In etch-and-rinse adhesives, the process of interface formation depends on the conditioning of the surface with phosphoric acid for its subsequent impregnation with the primer and adhesive. ^25^ ^,^ ^26^ The demineralized layer on the substrate surface is relatively deep and allows the formation of long resin tags inside the dentin tubules as well as greater penetration of the adhesive into the dentin bulk to produce a thick hybrid layer ^2^ ( Figure 2 ). However, the effect of the formed resin tags on the DBS bond strength has not been clarified yet. The results obtained in this work are consistent with the data reported by Nakabayashi, et al. ^27^ (1991) who examined different bond interfaces and concluded that although the lengths of the produced tags were very close, higher bond strengths were observed for the interfaces containing thicker hybrid layers, suggesting that the formation of tags had very little effect on the DBS bond strength.

When self-etch adhesives are used, the demineralization and infiltration of the monomer into the dentin layer occur simultaneously. ^28^ As a result, the smear layer is not removed and becomes incorporated into the hybrid layer interacting with dentin in various forms and at different depths depending on the acidic potential of the applied primer/bond. ^29^ This type of DBS promotes the formation of a thinner but more homogeneous hybrid layer with shorter, thinner, and less numerous tags. ^30^ The initial morphological characteristics of DBS observed in this study are identical to those reported in the literature ( Figure 3 ). As the self-etch adhesive interacts with dentin by transferring disposable calcium species from the substrate to the functional monomers present in the DBS structure, it can be hypothesized that the use of high-temperature laser alters the mineral content or arrangement, which affect their interaction overtime. Although all the studied CSE groups exhibited reduced bond strengths after 12 months of water storage, the groups treated with the laser were affected more strongly.

One interesting observation is related to the reduction of organic content after the application of a diode laser to radicular dentin, which was reported by Lopes, et al. ^18^ (2016). As indicated by the results of Raman micro spectroscopy analysis, the intensities of collagen bands were notably reduced after laser treatment. This implication overtime is still unknown in this case and requires more detailed information.

The bond strength values obtained in this work are in good agreement with the literature data since non-simplified adhesives exhibit better clinical and laboratory performance and thus are considered a golden standard in the field of adhesion. ^31^ The poorer performance of simplified adhesives is related to their composition since the presence of both hydrophilic and hydrophobic monomers in the same step produces a bonding interface with a non-solvated hydrophobic resin coating and a hybrid layer behaving like a semipermeable membrane that allows water movement through the interface. ^32^

Regarding the time factor, all groups in this study demonstrated a decrease in the bonding strength, suggesting that laser irradiation did not improve their longevity. It should be noted that this strategy was evaluated for non-solvated systems, even other aspects are related.

The limitations of this work are related to the standardization of the irradiated areas. In order to achieve the total irradiation of the sticks, the specimens were standardized in the dentin center using the matrix with an area of 36 mm^2^. In this region, the presence of the pulp chamber can significantly influence the material performance; as a result, the data obtained in some tests were negatively affected, leading to higher standard deviation values. To resolve this problem, future studies will be performed at an amplitude of the irradiated area.

In order to develop a proper surface treatment procedure for each clinical situation, it is important to understand the effect of different strategies on the mechanical properties of DBS. This study was mainly focused on the potential use of high-power lasers for the irradiation of non-simplified DBS containing etch-and-rinse adhesives. For such systems, stable chemical bonding can be apparently achieved without any additives. Further studies will be conducted to determine the advantages of using diode and other high-power lasers for the improvement of the longterm durability of the dentin bond interface.

## Conclusion

The results of this study indicate that the application of a diode laser did not improve the bond strength of etch-and-rinse DBS after 12 months of water storage. However, SEM images showed better penetration of the adhesives and formation of larger tags.
